# Automated verification of source dwell position in lunar ovoid applicators

**DOI:** 10.1002/acm2.70887

**Published:** 2026-10-30

**Authors:** Miquel Gubau‐Besalú, Adrià Casamitjana, Cristian Candela‐Juan, Eduard Agusti‐Camprubi, Gislè Segura‐Roca, Gerard Trias, Antonio Herreros

**Affiliations:** ^1^ University of Barcelona Barcelona Spain; ^2^ Computer Vision and Robotics Institute University of Girona Girona Spain; ^3^ Medical Physics Section Radiation Oncology Department Hospital Clínic Barcelona Barcelona Spain; ^4^ Radiation Protection Department Hospital Clínic Barcelona Barcelona Spain; ^5^ Infrastructure Management Hospital Clínic Barcelona Barcelona Spain; ^6^ Laboratory of Translational Genomics and Targeted therapies in Solid Tumors Institut d'Investigacions Biomèdiques August Pi i Sunyer Barcelona Spain

**Keywords:** AGA, brachytherapy, lunar ovoids, ring reconstruction, source path, Venezia applicator

## Abstract

**Background:**

Accurate reconstruction of the source dwell positions in high‐dose‐rate (HDR) brachytherapy is essential for treatment quality. The Venezia™ gynaecological applicator, with its lunar‐shaped ovoids, poses challenges because conventional dummy markers do not match the actual source path, introducing systematic uncertainties.

**Purpose:**

The aim of this work was to develop and demonstrate an automated, reproducible, and open‐source software for autoradiographic verification of dwell positions in Venezia lunar ovoids, and to compare measured positions with manufacturer‐provided applicator library data. This pipeline established a standardized approach for clinical commissioning and quality assurance (QA) workflows.

**Methods:**

A custom 3D‐printed holder was designed to standardize autoradiograph acquisition using radiochromic films. An open‐source Python pipeline, referred to as SPICA (Source Position Identification in Circular Applicators), was developed to process scanned films, isolate dwell position, and compute their coordinates with associated uncertainties. The code allows direct comparison between measured positions and manufacturer‐provided applicator library data. We performed preliminary validation of the software by measuring the trajectory of an Ir‐192 source for the 22 and 26 mm ovoids with a step size of 2.5 mm.

**Results:**

Mean discrepancies between measured and reference positions were 1.2 ± 0.5 and 1.5 ± 0.4 mm for the 22 and 26 mm ovoids, respectively, with a maximum deviation of 2.6 mm. Distal positions showed better agreement. These findings confirm that, in the tested applicator‐afterloader combination, vendor libraries provide a generally reliable baseline but may exhibit systematic differences.

**Conclusions:**

The proposed methodology offers a practical, reproducible solution to improve source path verification while reducing manual intervention. It supports more robust commissioning and QA of complex applicators and using the coordinates calculated in the treatment planning software can potentially reduce geometric uncertainties in clinical cases. Individualized commissioning remains essential for applicators with complex geometries.

## 1 INTRODUCTION

The Venezia™ gynaecological applicator (Elekta, Veenendaal, The Netherlands) was developed for treating cervical cancer with high‐dose‐rate (HDR) brachytherapy, allowing a hybrid combination of vaginal‐perineal interstitial and intracavitary components. This applicator is composed of a tandem, lunar‐shaped ovoids with source path diameters of 22, 26, and 30 mm, guides for parallel and oblique needles, and an optional perineal template.^1^

Dummy markers facilitate precise applicator reconstruction in radiation therapy planning. Elekta provides specialized x‐ray catheter sets and magnetic resonance (MR) markers for its gynaecological applicators, allowing source dwell positions to be identified directly on computed tomography (CT), x‐ray, or MR images. This method is particularly effective for low‐curvature applicators, where the alignment between the source path and the dummy marker is optimal.

Despite compatibility with the Venezia applicator, the provided dummy markers were not specifically designed for the lunar ovoid geometry, and the true source trajectory does not coincide with the markers or the lumen centerline due to the cable‐driven afterloader system and the large inner lumen diameter.^2^ Elekta therefore recommends the use of Applicator Library Files (ALF),^3^ which are virtual applicator models based on manufacturer measurements^4^, ^5^ that can be imported into Oncentra Brachy for source path reconstruction in the treatment planning system (TPS).

The ALF‐based reconstruction involves two steps: identification of the source path during commissioning and annual QA,^6^, ^7^, ^8^ and matching patient's anatomy with the applicator and source paths. The first step is highly sensitive to systematic errors, which can carry severe dosimetric consequences. For instance, a mere 1 mm dwell position displacement causes local dose variations of up to 25% in the vaginal mucosa.^2^, ^9^ Furthermore, systematic shifts of 2.5 mm can alter dose‐volume histogram (DVH) parameters by up to 3% for organs at risk and 1.2% for high‐risk clinical target volume (HR‐CTV) coverage.^10^

For this reason, the lunar ovoids of the Venezia applicator must be accurately commissioned before use in clinical settings to ensure that the source path indicated in the reconstruction step is correct. Several studies have reported discrepancies between measured dwell positions with applicator library values.^2^, ^11^, ^12^ This highlights the need to thoroughly characterize the actual source path within the specific afterloader–applicator combination. Measuring the real path is essential for the quantification of dosimetric variations relative to standard libraries prior to clinical use of the Venezia applicator.

This study proposes two novel tools for commissioning and QA of the Venezia applicator using autoradiographs: (i) a 3D printable phantom to attach the lunar ovoids; and (ii) a script to analyze the obtained images. The software gives a practical and open‐source solution for accurate source positioning verification.

## 2 MATERIALS AND METHODS

### 2.1 Venezia applicator

Venezia has available lunar‐shaped ovoids of three different sizes, with a circular source path of diameters (d) 22, 26, and 30 mm. The height (h) of the path in the ovoids was taken from the manufacturer as *h* = 6 mm and verified measuring the elevation of the lumen in CT images for the 22 and 26 mm ovoids. Dummy markers from the applicator vendor, which can be used to reconstruct the source path using radiographs or CT acquisitions, are shown with schemes of the applicator in Figure 1. The 30 mm ovoid was not available at our institution as it is not commonly used in clinical workflows in our partner institutions.

**FIGURE 1:**
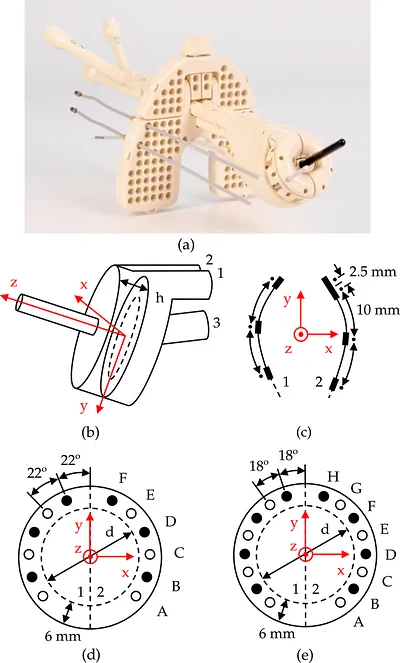
Schemes of Venezia™ applicator and dummy source markers, not to scale. The coordinate system proposed by Elekta^4^, ^5^ is embedded to facilitate the elements orientation, as well as the channel number of the ovoids. (a) The Venezia™ applicator as depicted in the user manual.^1^ (b) General view of the applicator with the plane of the lumen highlighted in dashed lines. (c) Dummy markers, where the distances between markers have been taken with respect to their centroids, indicated with a point. The first marker of the second channel was doubled to facilitate differentiation of the two catheters. (d) Ovoids of d = 22 mm. (e) Ovoids of d = 26 and 30 mm. The holes for the interstitial needles parallel to the intrauterine tube are filled with black, otherwise the white filled correspond to the oblique ones.

### 2.2 Holder design and setup to evaluate the source positioning

For reproducible positioning of the applicator on film we designed and manufactured a 3D‐printed holder to fix the applicator position relative to a radiochromic film (Figure 2). The holder was designed for use with the 0 mm intrauterine tube, which serves as mechanical fixation of the two lunar ovoids. Two pins coinciding with the ovoid holes were added for immobilization (Figure 2c). Exposures were acquired using Gafchromic™ RTQA2 films (Ashland, Bridgewater, NJ, USA), trimmed with a 5 cm squared die‐cutting machine.

**FIGURE 2:**
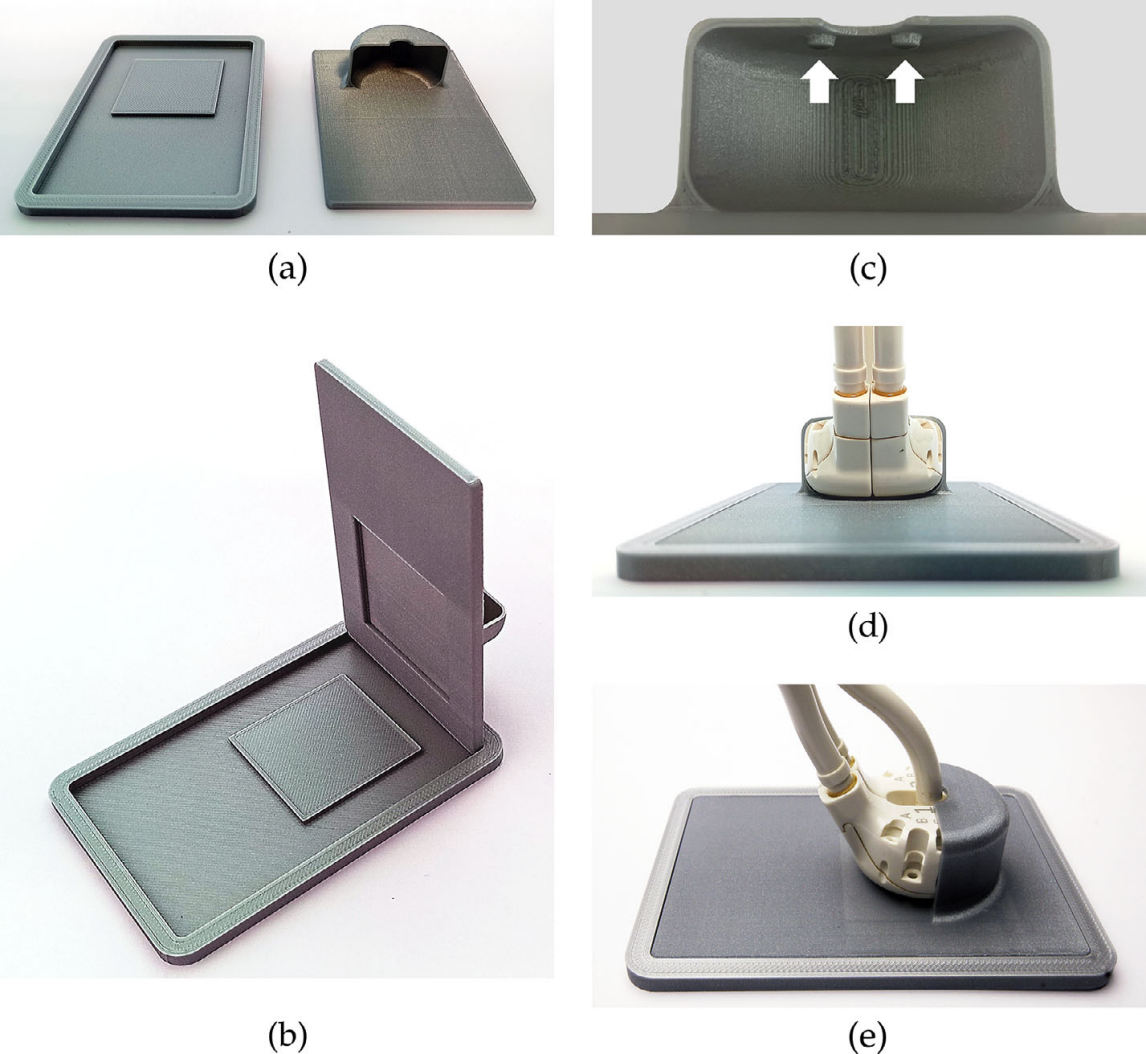
Photos of the 3D holder for the 22 mm ovoids. (a) and (b) show the two pieces with the film support cavity and cover. (c) White arrows show the two pins coincident with the central holes of the lunar ovoids. (d) and (e) show the Venezia™ applicator inside the designed holder.

The holder design followed five sequential steps^1^: measurement of the applicator external shape using a calliper (precision 0.05 mm)^2^; geometric modelling in Autodesk Fusion 360 (Mill Valley, CA, USA) with tolerances of 0.15–0.25 mm;^3^ prototyping with an Ultimaker S3 printer (Utrecht, The Netherlands) and functional validation; (4) iterative refinement of design and geometry; and (5) production of the final design, whose fabrication parameters and printable files are provided in Supplementary Materials (Table S1 and additional resources, respectively).

A radiograph of the applicator was obtained with a Gulmay D3100 kilovoltage unit (Xstrahl, Walsall, United Kingdom) to establish the x‐y coordinate origin at the ovoid center. The center was estimated from the applicator contour and refined using the centroid of the interstitial needle holes. To minimize beam divergence effects — arising because reference elements are elevated relative to the film plane — the x‐ray beam was aligned perpendicularly to the film using a perpendicularity test, with the tube positioned as far from the film as possible. The radiographs used for this purpose are available in Supplementary Materials.

Source path measurements were performed using the microSelectron v3 Digital afterloader with the HDR microSelectron v2 Ir‐192 source (both Elekta, Veenendaal, The Netherlands). After evaluating the feasibility of isolating multiple source positions on a single film, we opted to irradiate a single dwell position per film sheet. For Gafchromic radiochromic RTQA2 film and a source strength of 10 Ci, an exposure time of approximately 20 s per dwell position was required, resulting in total irradiation times of 140 and 160 s for the QA of the 22 and 26 mm ovoids, respectively. Although this approach increased the overall irradiation time, it reduced the uncertainties associated with signal superposition observed when multiple dwell positions were exposed on the same film. Using the more sensitive Gafchromic LD‐V1 film (Ashland, Bridgewater, NJ, USA), the required exposure times could be reduced by approximately one order of magnitude.

### 2.3 Analysis of the radiochromic films

The radiochromic films were digitized with an Epson Expression 12000XL (Seiko Epson Corp., Japan) scanner at a resolution of 300 dots per inch (dpi). Consistent film orientation and straightness were ensured by aligning each film to the scanner corner, making this a simple and reproducible step requiring no additional equipment (Figure 3a,c). A margin of no specific size had to be left around the reference corner (*rc*) to accurately match the radiochromic films. Then, the software trimmed them to the same dimensions. The images were horizontally inverted and a default common reference system, *x’‐y’*, was taken by the software.

**FIGURE 3:**
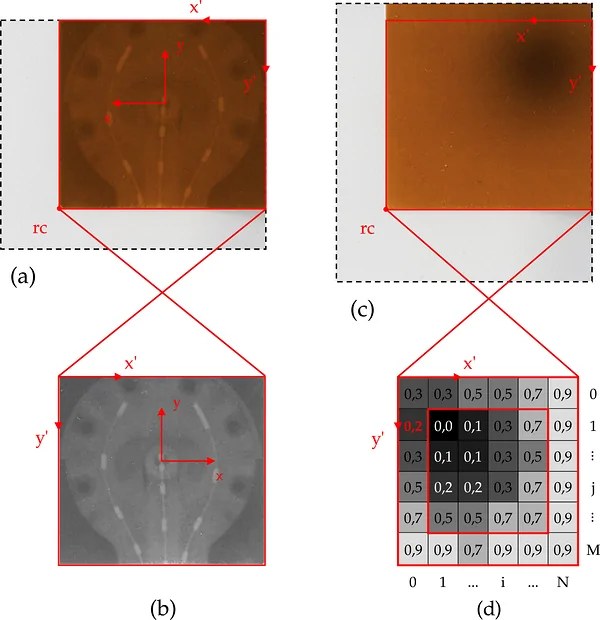
Example of the radiochromic films for the 22 mm diameter ovoids. (a) and (c) show the radiograph of the applicator and autoradiograph of the most distal position, (1500 mm) for the first channel, respectively. The scanned area is delimited by the dashed line. The software uses a reference corner (rc) of the films to match all images. The analyzed area is delimited in red, and it is defined considering the dimensions of all images. The reference system used by the software is x’‐y’. (b) shows the red channel of the scanned radiograph and (d) shows a sketch of the autoradiograph, both already trimmed and inverted. The threshold found to isolate the dose spots (inner red contour) was obtained selecting the perimeter lowest value.

To process the radiochromic films, a previous comparison of the histograms for the red, green and blue (RGB) color channels was done. The red one was selected for its better sensitivity (Figure 3b). Pixel values were normalized, ranging between 0 and 1, and a low‐pass filter was applied to reduce the grainy noise. Differential attenuation caused by thickness variations across the lunar ovoids and the surrounding distal holder cap (Figure 2e) introduced intensity gradients in the acquired radiographs (Figure 3b). A gradient mask was implemented to compensate for these image artifacts.

We encapsulated all steps into the open‐source software SPICA (Source Position Identification in Circular Applicators) due to its potential to verify circular applicators beyond Venezia. This is available in GitHub (https://github.com/acasamitjana/SPICA/). The code was programmed in Python v3.10.12, Python Software Foundation (2024), with clear usage instructions, documentation and examples.

### 2.4 Lunar ovoids’ center calculation

To obtain an approximation of the origin of the *x‐y* coordinate system, the ovoids were isolated by thresholding. The average value of the darkest pixels, which correspond to the background, was taken as a threshold and their centroid was calculated. Using this approximation, a mask comprising two concentric circles was applied to isolate the needle holes from surrounding regions. The holes parallel to the intrauterine tube were then used to refine the estimated center of the lunar ovoids through a two‐step procedure: first, by fitting a circle to the detected hole positions and minimizing its distance to their centroid; and second, by comparing the computed hole positions against the theoretical coordinates provided by the manufacturer and applying corrections where necessary.

### 2.5 Reconstruction of the source path

To clearly see the dose spots in the autoradiographs, a threshold filter was applied. A region of interest was defined to encompass all source positions for analysis. Because the radiation darkens the radiochromic films, the contour lowest value was taken as threshold (Figure 3d). Values below the threshold were used to identify the dwell locations x, calculated as the weighted mean:

(1) $$x=\frac{\sum_{i}w_{i}x_{i}}{\sum_{i}w_{i}}=\frac{\sum_{i}w_{i}x_{i}}{w}=\sum_{i}\;p_{i}x_{i},$$

where *x_i_* are the *i*‐th pixel 2D coordinates, *w_i_* is the corresponding weight modelled as the inverse intensity value (*1‐v_i_*, with *v_i_* being the min‐max normalized image intensity) and *p_i_* are the normalized probabilities $p_{i}=w_{i}\;/w$ with the normalization constant $w=\sum_{i}\;w_{i}$.

To evaluate the reliability of our measurements, we calculate the distance between the manufacturer's path model and the detected dwell positions as

(2) $$d=\sqrt{{\left(x-x_{0}\right)}^{2}}||x-x_{0}\left|\right|$$

where, x, $x_{0}$ are the 2D coordinates obtained from the autoradiographs and provided by the manufacturer, respectively.

### 2.6 Uncertainties

Type A uncertainty was assessed through statistical analysis of the dose spots’ shape. This uncertainty represents random variability in the evaluation process. The uncertainty associated with centroid calculation ($u_{A}^{th}$) arises from the dispersion of pixel positions used to determine the dwell location on autoradiographs. This variability depends on image quality and the thresholding accuracy. This centroid uncertainty is given by the standard deviation of the source centroid coordinates, $u_{A}^{th}=\sigma_{th}$ calculated as

(3) $$\sigma_{th}=\sqrt{\frac{1}{N_{px}}\sum_{i}p_{i}{\left(x_{i}-x_{0}\right)}^{2},}\;$$

where x, $x_{i}$ and $p_{i}$ are given by Equation 1, and $N_{px}$ is the number of thresholded pixels used to calculate each centroid.

Type B uncertainties represent systematic effects from four sources: scanner resolution, which determines the minimum detectable displacement; geometric tolerances of the 3D‐printed holder; beam divergence, accounting for potential misalignment between the x‐ray beam axis and the ovoid center (tube elevation: 1400 mm); and the afterloader positional uncertainty of 1 mm. All components were combined using the root‐sum‐square method to estimate the overall measurement uncertainty.

## 3 RESULTS

The source paths for the Venezia applicator with 22 and 26 mm diameter ovoids and a step size of 2.5 mm were evaluated and shown in Figure 4a,b, aiming to replicate the initial commissioning process. The measurements were taken from a single applicator on different sets of radiochromic films with a single exposure per position. We qualitatively compared the calculated dwell positions (either single measurements or the mean between multiple repetitions if available) and the positions provided by the manufacturer. These theoretical positions for each ovoid diameter are appended in the Supplementary Materials (Tables S2 and S3). Furthermore, a typical QA scenario is depicted in Figure 4c,d, where multiple exposures are recorded at 4 different positions per channel. The median number of repetitions is 8 per channel (complete details in Supplementary Materials, Table S5). For all Figure 4a–d), we also show the theoretical and computed interstitial hole positions for all autoradiographs available, for reference. The presence of multiple interstitial holes in each direction inherently mitigates the effect of individual misdetections, and robustness is further improved as the multiple acquisitions are available.

**FIGURE 4:**
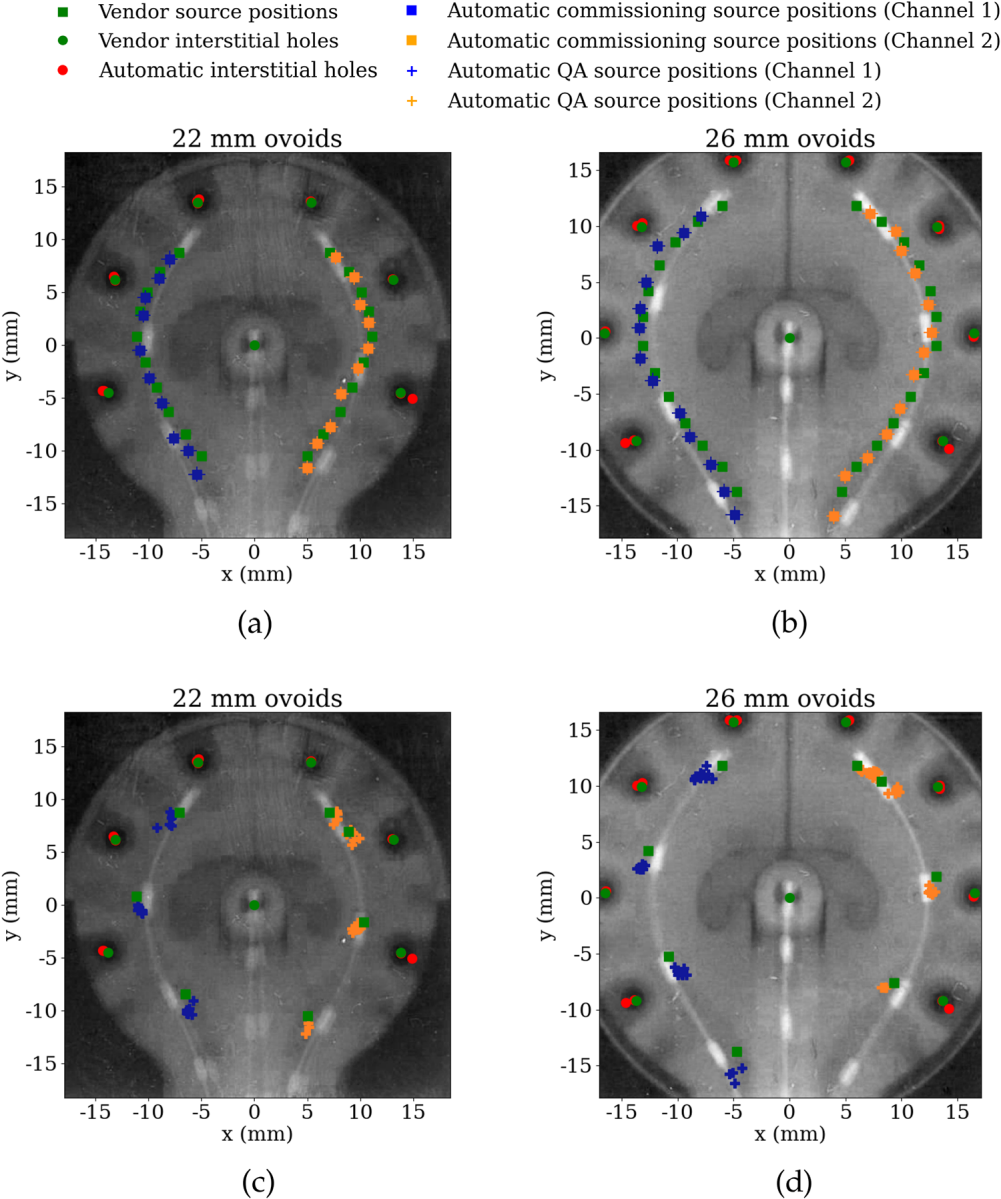
Software output images of the (a, c) 22 mm and (b, d) 26 mm diameter ovoids. We show the commissioning scenario, with measurements every 2.5 mm steps (a, b) and the QA scenario with multiple repeated exposures (c, d). Squares are used to depict source dwell positions. Green ones correspond to the positions included in the manufacturer's libraries, whereas the blue squares are used for positions obtained from processed autoradiographs of channel 1 and the orange squares for channel 2. Crosses correspond to processed autoradiograph repetitions, with uncertainties smaller than the symbols. We also show the interstitial holes as computed from radiographs (red circles) and their theoretical locations provided by the manufacturer (green circles). The calculated centers of the ovoids are also highlighted with a green circle and the coordinate system used is the one specified in Figure 1.

The discrepancies between coordinates detected from autoradiographs using the SPICA software and the theoretical coordinates provided by the manufacturer's libraries^4^, ^5^ are shown in Figure 5 for both the 22 and 26 mm diameter ovoids. The uncertainty of our measurements is depicted using error bars. The mean discrepancy across all dwell positions is 1.2 ± 0.5 mm for 22 mm ovoids and 1.5 ± 0.4 mm for 26 mm. The exact mean discrepancy values among all repetitions for different channels and positions can be found in the Supplementary Materials (Table S4). We note that single measurements can have large discrepancies in isolation. In Supplementary Materials (Table S5), we provide the largest distance between any repeated measurement and the dwell positions provided by the manufacturer's libraries in different scenarios (channels, positions). These most distant repetitions can be considered as outliers from our method. As an example, excluding these measurements for channel 1 in the 22 mm ovoids, the mean discrepancy is reduced to 0.9 ± 0.9 mm.

**FIGURE 5:**
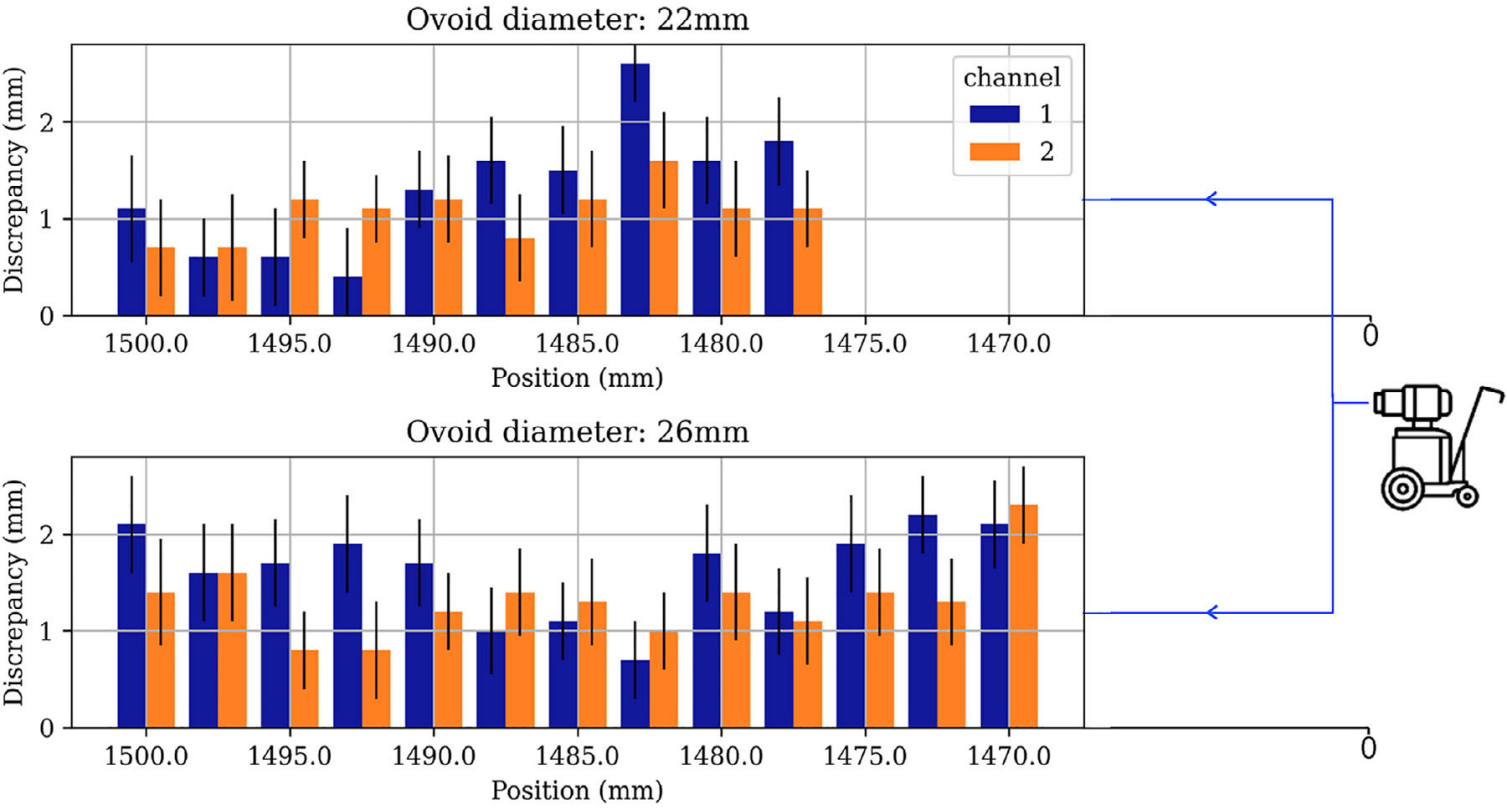
Discrepancies, d, between coordinates obtained from autoradiographs and manufacturer's libraries for the 22 mm (upper plot) and 26 mm (lower plot) ovoids. The x‐axis represents the distance at which the source is projected. The blue bars correspond to channel 1 and the orange bars to channel 2. The error bars represent the computed uncertainties.

To verify the step size configured in the afterloader, we calculated the distance between adjacent positions. Due to the small 2.5 mm step used, we opted for a linear approximation of the curved path. An average of these distances resulted in 2.4 ± 0.5 mm for the 22 mm ovoids and 2.5 ± 0.4 mm for 26 mm, which are consistent with the step programmed.

We calculated the uncertainty of all the positions, with an overall value of 0.7 mm (*k *= 1, where *k* is the coverage factor). A detailed stratification between different uncertainty types is reported in Table 1. The mean standard deviation of multiple exposure of the same dwell position was calculated as 0.38 mm, consistent with the source positioning uncertainty and only the latter was considered for the combined uncertainty.

**TABLE 1:** Summary of measurement uncertainties for dwell position evaluation in the lunar ovoids. Type A refers to statistical (random) uncertainties, while Type B corresponds to systematic uncertainties based on equipment specifications and setup conditions. The beam divergence was calculated assuming a maximum misalignment between the x‐ray beam centerline and the center of the ovoids equal to the radius of the ovoids.

	Uncertainty (mm)	
Uncertainty component	Type A	Type B
Centroid calculation	0.01	
Image resolution		0.08
Geometric holder tolerances		0.25
Beam divergence		0.16
Source positioning uncertainty		0.58
Total combined uncertainty (*k *= 1)	0.7	
Total combined uncertainty (*k *= 2)	1.4	

We also calculated the uncertainties of the discrepancies between the coordinates obtained from autoradiographs and the theoretical values. These uncertainties were propagated from the positions’ uncertainties according to Equation 2. Considering the position's uncertainty from Table 1, the overall distance's uncertainty becomes $0.7\cdot \;\sqrt{2}=1$ mm.

## 4 DISCUSSION

The visual discrepancy of our measurements with the radiographic markers is consistent with the intrinsic design of the Venezia applicator, where the curved channels induce subtle geometric offsets to source that the markers fail to capture. As a consequence, relying on dummy markers for reconstruction may introduce clinically relevant inaccuracies in dwell localization. The measurements obtained highlight this mismatch and support the need for independent verification methods based on autoradiography, rather than on radiographic markers alone. The use of virtual applicator libraries, as recommended by Elekta, provides a more reliable reference for clinical planning. However, even these libraries may present regular deviations that can potentially impact dosimetric calculations, underscoring the need for individualized commissioning.

The evaluation of the Venezia applicator's source path in our study has revealed systematic deviations between the measured dwell positions and those provided by the manufacturer's applicator libraries.^4^, ^5^ While the found discrepancies are typically lower than the 2.5 mm step size (mean value of 1.2 ± 0.5 mm and 1.5 ± 0.4 mm for the 22 and 26 mm ovoids, respectively), they can reach up to 2.6 mm in the worst case.

The largest discrepancies (d > *2 *mm) are mainly found in the most proximal positions, where the source enters the ovoids and the lumen rises. In this region, the autoradiographic intensity is notably reduced compared to distal positions resulting in poorer image detectability and centroid estimation accuracy. Proximal dwells exhibited a mean pixel intensity approximately 30–40% lower than those at distal locations. On the other hand, the most distal positions generally present greater agreement with the manufacturer's library positions, except for channel 1 in the 22 mm ovoids. Based on these observations, we recommend that practitioners implementing this methodology treat measured discrepancies exceeding 2 mm with additional caution, as they are more likely to reflect reduced image quality than true geometric deviations.

The results from this study are consistent with deviations previously reported in the literature for curved applicators, including the Venezia.^2^, ^11^, ^12^, ^13^. One of the benefits from our method is that it uses the real HDR source, in contrast with other previous studies, such as Hansen et al,^2^ where they used the Flexitron^®^ source position simulator (SPS) as a surrogate. They compared several reconstruction models with the SPS and the best fit corresponded to the Elekta model, with average discrepancies of 0.7‐1.0 mm for all planning positions across all three applicator sizes. The disagreement of the most distal positions was measured around 0.3‐0.9 mm. Li et al.^13^ studied the source path length (SPL), proposed by Awunor et al,^14^ between adjacent positions. To do this, they sent the SPS at different positions with a step of 10 mm. Using three different methods and two distinct programs, they obtained measurements between 9.8 and 10.0 mm. Unlike methods relying on source position simulators, the approach presented here uses the actual HDR source, more closely replicating a real clinical commissioning or QA scenario.

Other imaging modalities were also explored in the literature. The work from Kihara et al.^12^ used x‐ray fluoroscopy to study two different sets of Venezia applicators. Their measurements show a mean discrepancy with the planned positions across all dwell positions and tested ovoids of 0.4 mm in x‐ and y‐directions, and a maximum values of 0.8 mm and 0.9 mm for the x‐ and y‐direction, respectively. Also, the differences in the most distal position range from 0.3 to 0.8 mm depending on the set and the ovoid. A novel technique using 2D optical imaging was recently proposed by Borg et al.^11^ They found average discrepancies of 0.5 mm in both applicator sizes between the planned and the measured positions. The maximum discrepancy was 1.3 mm for the 22 mm ovoids and 0.7 mm for the 26 mm. One limitation of Borg et al.^11^ methodology is the assumption that all dwell positions lie within a single plane parallel to the scintillation film. Under this assumption, their system cannot distinguish between vertical and lateral displacements. Because of the lumen radius, the vertical displacement would produce a 0.2 mm horizontal displacement in the film. Our method offers a simpler and straightforward solution, eliminating signal distortions and displacements caused by vertical source movement. Another study from Li et al. [13] point out that the design of Venezia in the proximal dwell positions has created difficulties in accurately determining the source location. This is due to the insertion angle and the fact that the source moves out of the 2D plane defined by the other dwell positions. The similar studies consulted had clinical tolerances of 1 mm,^2^, ^11^, ^12^, ^13^ and Borg et al.^11^ had an action level of 2 mm.

The tools provided here, including the files to generate 3D printed holders, and the SPICA software to automatically analyze the results, can be used to facilitate a robust and reproducible QA of source positions within the applicator using autoradiographs. Nonetheless, we note that the software development and validation experiments were performed using the triplet microSelectron v2 source, microSelectron v3 Digital afterloader and the corresponding Venezia applicator libraries. Consequently, the SPICA pipeline and the workflow presented here are optimized for the mechanical and geometric characteristics of this specific system. Although the underlying methodology is generalizable, further work is required to implement and validate the analysis for the libraries of the Flexitron® afterloader and its corresponding source design, whose cable mechanics and source offset differ from the microSelectron and may introduce systematic variations in dwell positioning.

One of the limitations of this work is that the verification of dwell positions is inherently planar: autoradiographs capture the projection of the source onto the film plane corresponding to the lumen plane of the lunar ovoids but provide no information in the perpendicular (out‐of‐plane) direction. As a result, any deviations related to the elevation of the source within the lumen, particularly relevant in the proximal region where the path rises, cannot be quantified with the current approach. Incorporating a perpendicular film strip around the lunar ovoids is necessary to overcome these constraints and left as future work.

A further limitation of this study is that it does not include a dedicated dosimetric analysis of the impact of the observed source path deviations on clinical dose distributions. While geometric discrepancies of up to 2–3 mm were identified between measured dwell positions and manufacturer‐provided libraries, their direct dosimetric consequences were not evaluated within patient treatment plans. Nevertheless, previous studies have shown that source path shifts of this magnitude typically result in limited dosimetric effects. In particular, previous studies^10^ reported that systematic and random shifts of ± 2.5 mm applied to all dwell positions led to variations of less than 3% in D_2_ cm^3^ for bladder, rectum, and sigmoid, while the impact on DVH parameters for the HR‐CTV and gross tumor volume (GTV) was considerably smaller. These findings suggest that the deviations observed in the present work are unlikely to result in clinically significant dose changes, although a dedicated patient‐specific dosimetric evaluation would be required to formally confirm this assumption.

## 5 CONCLUSION

This study addresses the challenging evaluation of the Venezia HDR brachytherapy applicator using autoradiographs, offering a reproducible and adaptable solution. The proposed approach combines a custom‐designed 3D‐printed holder and an automated image analysis script, both of which are made openly available to the community. These tools aim to standardize the acquisition process and reduce manual intervention during the evaluation of autoradiographs, automating the results processing and comparison with reference positions.

The results demonstrate that the discrepancies between the measured source positions and the manufacturer's references can reach up to 2.6 mm, particularly in the proximal regions of the ovoids, where the lumen elevation affects the visibility and accuracy of the dose spots in the autoradiographs. In contrast, the distal positions show better agreement. The observed deviations underscore the importance of individualized commissioning for each applicator and size.

## AUTHOR CONTRIBUTIONS

Conceptualization: Cristian Candela‐Juan, Antonio Herreros Methodology: Miquel Gubau‐Besalú, Cristian Candela‐Juan, Eduard Agusti‐Camprubi, Gislè Segura Roca, Gerard Trias Antonio Herreros Software: Miquel Gubau‐Besalú, Adrià Casamitjana Validation: Miquel Gubau‐Besalú, Adrià Casamitjana Formal Analysis: Miquel Gubau‐Besalú Investigation: Eduard Agusti‐Camprubi Resources: Gislè Segura Roca, Gerard Trias Writing—original draft: Miquel Gubau‐Besalú, Adrià Casamitjana, Cristian Candela‐Juan, Antonio Herreros Writing—review & editing: Miquel Gubau‐Besalú, Adrià Casamitjana, Cristian Candela‐Juan, Antonio Herreros Supervision: Cristian Candela‐Juan, Antonio Herreros.

## CONFLICT OF INTEREST STATEMENT

The authors declare no conflicts of interest.

## ETHICS STATEMENT

This project is determined to be a Quality Assurance initiative that does not constitute human subjects research. Consequently, formal IRB approval and informed consent were not required.

## Supporting information




Supporting Information

Supporting Information

Supporting Information

Supporting Information

Supporting Information

Supporting Information

Supporting Information

## Data Availability

The stereolithography files are available online (https://www.printables.com/model/1650955‐support‐venezia). The complete set of autoradiographs and radiographs can be made available upon reasonable request to the corresponding author.
