# Supplementary material for: Automated verification of source dwell position in lunar ovoid applicators
Source: J Appl Clin Med Phys. 2026 Oct 30;27(11):e70887. doi: 10.1002/acm2.70887 (PMC13722779; doi:10.1002/acm2.70887)
Supplement: Supplementary file 1 [file ACM2-27-e70887-s001.docx]

**Supplementary Materials**

**Table 1.** Fabrication parameters for the design of the Venezia^TM^ holder.

| **Settings for the 3D printer** | |
| --- | --- |
| Infill density | 20 % |
| Shell thickness | 0.8 mm |
| Layer height | 1.0 mm |
| Material | Generic PLA |
| Total print time | 2 hours 44 minutes |
| Filament amount | 18 g |
| Support material | Yes |
| Support type | Tree |
| Support placement | If possible, in non-functional surfaces |

**Table 2.** Coordinates of the source in the 22 mm ovoids, the afterloader channel and position are specified.

| **Afterloader channel** | **Position (mm)** | **x (mm)** | **y (mm)** |
| --- | --- | --- | --- |
| 1 | 1500.0 | -8.0 ± 0.7 | 8.1 ± 0.7 |
| 1 | 1497.5 | -9.0 ± 0.7 | 6.3 ± 0.7 |
| 1 | 1495.0 | -10.3 ± 0.7 | 4.5 ± 0.7 |
| 1 | 1492.5 | -10.5 ± 0.7 | 2.8 ± 0.7 |
| 1 | 1490.0 | -10.8 ± 0.7 | -0.5 ± 0.7 |
| 1 | 1487.5 | -9.9 ± 0.7 | -3.1 ± 0.7 |
| 1 | 1485.0 | -8.7 ± 0.7 | -5.5 ± 0.7 |
| 1 | 1482.5 | -7.6 ± 0.7 | -8.8 ± 0.7 |
| 1 | 1480.0 | -6.2 ± 0.7 | -10.0 ± 0.7 |
| 1 | 1477.5 | -5.4 ± 0.7 | -12.2 ± 0.7 |
| 2 | 1500.0 | 7.7 ± 0.7 | 8.3 ± 0.7 |
| 2 | 1497.5 | 9.4 ± 0.7 | 6.4 ± 0.7 |
| 2 | 1495.0 | 10.0 ± 0.7 | 3.8 ± 0.7 |
| 2 | 1492.5 | 10.8 ± 0.7 | 2.1 ± 0.7 |
| 2 | 1490.0 | 10.7 ± 0.7 | -0.3 ± 0.7 |
| 2 | 1487.5 | 9.8 ± 0.7 | -2.2 ± 0.7 |
| 2 | 1485.0 | 8.2 ± 0.7 | -4.6 ± 0.7 |
| 2 | 1482.5 | 7.2 ± 0.7 | -7.7 ± 0.7 |
| 2 | 1480.0 | 5.9 ± 0.7 | -9.3 ± 0.7 |
| 2 | 1477.5 | 5.0 ± 0.7 | -11.6 ± 0.7 |

**Table 3.** Coordinates of the source in the 26 mm ovoids, the afterloader channel and position are specified.

| **Afterloader channel** | **Position (mm)** | **x (mm)** | **y (mm)** |
| --- | --- | --- | --- |
| 1 | 1500.0 | -7.9 ± 0.7 | 10.9 ± 0.7 |
| 1 | 1497.5 | -9.4 ± 0.7 | 9.4 ± 0.7 |
| 1 | 1495.0 | -11.8 ± 0.7 | 8.2 ± 0.7 |
| 1 | 1492.5 | -12.8 ± 0.7 | 5.0 ± 0.7 |
| 1 | 1490.0 | -13.3 ± 0.7 | 2.6 ± 0.7 |
| 1 | 1487.5 | -13.4 ± 0.7 | 0.9 ± 0.7 |
| 1 | 1485.0 | -13.3 ± 0.7 | -1.8 ± 0.7 |
| 1 | 1482.5 | -12.2 ± 0.7 | -3.8 ± 0.7 |
| 1 | 1480.0 | -9.8 ± 0.7 | -6.7 ± 0.7 |
| 1 | 1477.5 | -8.9 ± 0.7 | -8.8 ± 0.7 |
| 1 | 1475.0 | -7.0 ± 0.7 | -11.3 ± 0.7 |
| 1 | 1472.5 | -5.8 ± 0.7 | -13.7 ± 0.7 |
| 1 | 1470.0 | -4.9 ± 0.7 | -15.8 ± 0.7 |
| 2 | 1500.0 | 7.2 ± 0.7 | 11.1 ± 0.7 |
| 2 | 1497.5 | 9.5 ± 0.7 | 9.5 ± 0.7 |
| 2 | 1495.0 | 10.0 ± 0.7 | 7.8 ± 0.7 |
| 2 | 1492.5 | 11.2 ± 0.7 | 5.8 ± 0.7 |
| 2 | 1490.0 | 12.4 ± 0.7 | 3.0 ± 0.7 |
| 2 | 1487.5 | 12.7 ± 0.7 | 0.5 ± 0.7 |
| 2 | 1485.0 | 12.0 ± 0.7 | -1.3 ± 0.7 |
| 2 | 1482.5 | 11.1 ± 0.7 | -3.3 ± 0.7 |
| 2 | 1480.0 | 9.9 ± 0.7 | -6.3 ± 0.7 |
| 2 | 1477.5 | 8.7 ± 0.7 | -8.6 ± 0.7 |
| 2 | 1475.0 | 7.0 ± 0.7 | -10.7 ± 0.7 |
| 2 | 1472.5 | 5.0 ± 0.7 | -12.3 ± 0.7 |
| 2 | 1470.0 | 4.0 ± 0.7 | -15.9 ± 0.7 |

**Table 4.** Discrepancies, d, between coordinates obtained from autoradiographs and manufacturer’s libraries for the 22 and 26 mm ovoids, corresponding to the positions displayed in Fig. 4 (a, b) from the main manuscript. The afterloader channel and position are also included.

|  | **22 mm ovoids** | | **26 mm ovoids** | |
| --- | --- | --- | --- | --- |
|  | **Afterloader channel 1** | **Afterloader channel 2** | **Afterloader channel 1** | **Afterloader channel 2** |
| Position (mm) | d (mm) | d (mm) | d (mm) | d (mm) |
| 1500.0 | 1.1 ± 0.9 | 0.7 ± 0.9 | 2.1 ± 0.9 | 1.4 ± 0.9 |
| 1497.5 | 0.6 ± 0.7 | 0.7 ± 0.9 | 1.6 ± 0.9 | 1.6 ± 0.9 |
| 1495.0 | 0.6 ± 0.9 | 1.2 ± 0.7 | 1.7 ± 0.8 | 0.8 ± 0.8 |
| 1492.5 | 0.4 ± 0.9 | 1.1 ± 0.7 | 1.9 ± 0.9 | 0.8 ± 0.9 |
| 1490.0 | 1.3 ± 0.8 | 1.2 ± 0.8 | 1.7 ± 0.9 | 1.2 ± 0.8 |
| 1487.5 | 1.6 ± 0.8 | 0.8 ± 0.9 | 1.0 ± 0.8 | 1.4 ± 0.8 |
| 1485.0 | 1.5 ± 0.8 | 1.2 ± 0.9 | 1.1 ± 0.7 | 1.3 ± 0.9 |
| 1482.5 | 2.6 ± 0.8 | 1.6 ± 0.9 | 0.7 ± 0.8 | 1.0 ± 0.8 |
| 1480.0 | 1.6 ± 0.8 | 1.1 ± 0.9 | 1.8 ± 0.9 | 1.4 ± 0.9 |
| 1477.5 | 1.8 ± 0.8 | 1.1 ± 0.7 | 1.2 ± 0.8 | 1.1 ± 0.9 |
| 1475.0 |  |  | 1.9 ± 0.9 | 1.4 ± 0.9 |
| 1472.5 |  |  | 2.2 ± 0.7 | 1.3 ± 0.9 |
| 1470.0 |  |  | 2.1 ± 0.7 | 2.3 ± 0.8 |

**Table 5.** Distances between the manufacturer’s libraries and the most distant autoradiograph centroid, $d_{max}$, in the case of multiple exposures depicted in Fig. 4 (c, d) from the main manuscript. The afterloader channel and position are also included.

|  |  | **22 mm ovoids** | | **26 mm ovoids** | |
| --- | --- | --- | --- | --- | --- |
| **Position (mm)** | **Afterloader channel** | $d_{max}$ **(mm)** | Num. exposures | $d_{max}$ **(mm)** | Num. exposures |
| 1500.0 | 1 | 2.5 ± 1.1 | 8 | 2.9 ± 1.1 | 13 |
| 1490.0 | 1 | 1.6 ± 1.0 | 8 | 2.0 ± 1.1 | 8 |
| 1480.0 | 1 | 2.0 ± 1.0 | 6 | 2.2 ± 1.1 | 8 |
| 1470.0 | 1 |  |  | 2.9 ± 0.9 | 4 |
| 1500.0 | 2 | 1.3 ± 1.1 | 8 | 2.0 ± 1.0 | 14 |
| 1497.5 | 2 | 1.4 ± 1.1 | 7 | 1.9 ± 1.1 | 8 |
| 1487.5 | 2 | 1.2 ± 1.1 | 8 | 1.8 ± 0.9 | 8 |
| 1477.5 | 2 | 1.7 ± 0.8 | 3 | 1.3 ± 1.1 | 5 |

The uncertainties of Tables 4 and 5 were propagated from the positions’ uncertainties according to Eq. 1 from the main manuscript (and repeated here for convenience)

$$d=\left\| x-x_{0} \right\|,$$

where, $x, x_{0}$ are the 2D coordinates obtained from the autoradiographs and the ones provided by the manufacturer, respectively. Considering the position’s uncertainty from Table 1 of the main manuscript, the overall distance’s uncertainty becomes $0.7\cdot2/\sqrt{2}=1.1 mm$

**Additional resources**

- SPICA wepage: <https://github.com/acasamitjana/SPICA/>
- Stereolithography files: <https://www.printables.com/model/1650955-support-venezia>
- Radiographs used: <https://github.com/acasamitjana/SPICA/ANON/data/default_rads>
