# Supplementary material for: Automated verification of source dwell position in lunar ovoid applicators
Source: J Appl Clin Med Phys. 2026 Oct 30;27(11):e70887. doi: 10.1002/acm2.70887 (PMC13722779; doi:10.1002/acm2.70887)
Supplement: Supplementary file 2 [file ACM2-27-e70887-s002.docx]

**Table S-4.** Discrepancies, d, between coordinates obtained from autoradiographs and manufacturer’s libraries for the 22 and 26 mm ovoids, corresponding to the positions displayed in Fig. 4 (a, b) from the main manuscript. The afterloader channel and position are also included.

|  | **22 mm ovoids** | | **26 mm ovoids** | |
| --- | --- | --- | --- | --- |
|  | **Afterloader channel 1** | **Afterloader channel 2** | **Afterloader channel 1** | **Afterloader channel 2** |
| Position (mm) | d (mm) | d (mm) | d (mm) | d (mm) |
| 1500.0 | 1.1 ± 0.9 | 0.7 ± 0.9 | 2.1 ± 0.9 | 1.4 ± 0.9 |
| 1497.5 | 0.6 ± 0.7 | 0.7 ± 0.9 | 1.6 ± 0.9 | 1.6 ± 0.9 |
| 1495.0 | 0.6 ± 0.9 | 1.2 ± 0.7 | 1.7 ± 0.8 | 0.8 ± 0.8 |
| 1492.5 | 0.4 ± 0.9 | 1.1 ± 0.7 | 1.9 ± 0.9 | 0.8 ± 0.9 |
| 1490.0 | 1.3 ± 0.8 | 1.2 ± 0.8 | 1.7 ± 0.9 | 1.2 ± 0.8 |
| 1487.5 | 1.6 ± 0.8 | 0.8 ± 0.9 | 1.0 ± 0.8 | 1.4 ± 0.8 |
| 1485.0 | 1.5 ± 0.8 | 1.2 ± 0.9 | 1.1 ± 0.7 | 1.3 ± 0.9 |
| 1482.5 | 2.6 ± 0.8 | 1.6 ± 0.9 | 0.7 ± 0.8 | 1.0 ± 0.8 |
| 1480.0 | 1.6 ± 0.8 | 1.1 ± 0.9 | 1.8 ± 0.9 | 1.4 ± 0.9 |
| 1477.5 | 1.8 ± 0.8 | 1.1 ± 0.7 | 1.2 ± 0.8 | 1.1 ± 0.9 |
| 1475.0 |  |  | 1.9 ± 0.9 | 1.4 ± 0.9 |
| 1472.5 |  |  | 2.2 ± 0.7 | 1.3 ± 0.9 |
| 1470.0 |  |  | 2.1 ± 0.7 | 2.3 ± 0.8 |

**Table 5.** Distances between the manufacturer’s libraries and the most distant autoradiograph centroid, $d_{max}$, in the case of multiple exposures depicted in Fig. 4 (c, d) from the main manuscript. The afterloader channel and position are also included.

|  |  | **22 mm ovoids** | | **26 mm ovoids** | |
| --- | --- | --- | --- | --- | --- |
| **Position (mm)** | **Afterloader channel** | $d_{max}$ **(mm)** | Num. exposures | $d_{max}$ **(mm)** | Num. exposures |
| 1500.0 | 1 | 2.5 ± 1.1 | 8 | 2.9 ± 1.1 | 13 |
| 1490.0 | 1 | 1.6 ± 1.0 | 8 | 2.0 ± 1.1 | 8 |
| 1480.0 | 1 | 2.0 ± 1.0 | 6 | 2.2 ± 1.1 | 8 |
| 1470.0 | 1 |  |  | 2.9 ± 0.9 | 4 |
| 1500.0 | 2 | 1.3 ± 1.1 | 8 | 2.0 ± 1.0 | 14 |
| 1497.5 | 2 | 1.4 ± 1.1 | 7 | 1.9 ± 1.1 | 8 |
| 1487.5 | 2 | 1.2 ± 1.1 | 8 | 1.8 ± 0.9 | 8 |
| 1477.5 | 2 | 1.7 ± 0.8 | 3 | 1.3 ± 1.1 | 5 |

**Additional resources**

- SPICA wepage: <https://github.com/acasamitjana/SPICA/>
- Stereolithography files: <https://www.printables.com/model/1650955-support-venezia>
- Radiographs used: <https://github.com/acasamitjana/SPICA/ANON/data/default_rads>
