# Supplementary material for: Automated verification of source dwell position in lunar ovoid applicators
Source: J Appl Clin Med Phys. 2026 Oct 30;27(11):e70887. doi: 10.1002/acm2.70887 (PMC13722779; doi:10.1002/acm2.70887)
Supplement: Supplementary file 3 [file ACM2-27-e70887-s003.docx]

**Table S-2.** Coordinates of the source in the 22 mm ovoids, the afterloader channel and position are specified.

| **Afterloader channel** | **Position (mm)** | **x (mm)** | **y (mm)** |
| --- | --- | --- | --- |
| 1 | 1500.0 | -8.0 ± 0.7 | 8.1 ± 0.7 |
| 1 | 1497.5 | -9.0 ± 0.7 | 6.3 ± 0.7 |
| 1 | 1495.0 | -10.3 ± 0.7 | 4.5 ± 0.7 |
| 1 | 1492.5 | -10.5 ± 0.7 | 2.8 ± 0.7 |
| 1 | 1490.0 | -10.8 ± 0.7 | -0.5 ± 0.7 |
| 1 | 1487.5 | -9.9 ± 0.7 | -3.1 ± 0.7 |
| 1 | 1485.0 | -8.7 ± 0.7 | -5.5 ± 0.7 |
| 1 | 1482.5 | -7.6 ± 0.7 | -8.8 ± 0.7 |
| 1 | 1480.0 | -6.2 ± 0.7 | -10.0 ± 0.7 |
| 1 | 1477.5 | -5.4 ± 0.7 | -12.2 ± 0.7 |
| 2 | 1500.0 | 7.7 ± 0.7 | 8.3 ± 0.7 |
| 2 | 1497.5 | 9.4 ± 0.7 | 6.4 ± 0.7 |
| 2 | 1495.0 | 10.0 ± 0.7 | 3.8 ± 0.7 |
| 2 | 1492.5 | 10.8 ± 0.7 | 2.1 ± 0.7 |
| 2 | 1490.0 | 10.7 ± 0.7 | -0.3 ± 0.7 |
| 2 | 1487.5 | 9.8 ± 0.7 | -2.2 ± 0.7 |
| 2 | 1485.0 | 8.2 ± 0.7 | -4.6 ± 0.7 |
| 2 | 1482.5 | 7.2 ± 0.7 | -7.7 ± 0.7 |
| 2 | 1480.0 | 5.9 ± 0.7 | -9.3 ± 0.7 |
| 2 | 1477.5 | 5.0 ± 0.7 | -11.6 ± 0.7 |
