# Supplementary material for: Automated verification of source dwell position in lunar ovoid applicators
Source: J Appl Clin Med Phys. 2026 Oct 30;27(11):e70887. doi: 10.1002/acm2.70887 (PMC13722779; doi:10.1002/acm2.70887)
Supplement: Supplementary file 4 [file ACM2-27-e70887-s004.docx]

**Table S-3.** Coordinates of the source in the 26 mm ovoids, the afterloader channel and position are specified.

| **Afterloader channel** | **Position (mm)** | **x (mm)** | **y (mm)** |
| --- | --- | --- | --- |
| 1 | 1500.0 | -7.9 ± 0.7 | 10.9 ± 0.7 |
| 1 | 1497.5 | -9.4 ± 0.7 | 9.4 ± 0.7 |
| 1 | 1495.0 | -11.8 ± 0.7 | 8.2 ± 0.7 |
| 1 | 1492.5 | -12.8 ± 0.7 | 5.0 ± 0.7 |
| 1 | 1490.0 | -13.3 ± 0.7 | 2.6 ± 0.7 |
| 1 | 1487.5 | -13.4 ± 0.7 | 0.9 ± 0.7 |
| 1 | 1485.0 | -13.3 ± 0.7 | -1.8 ± 0.7 |
| 1 | 1482.5 | -12.2 ± 0.7 | -3.8 ± 0.7 |
| 1 | 1480.0 | -9.8 ± 0.7 | -6.7 ± 0.7 |
| 1 | 1477.5 | -8.9 ± 0.7 | -8.8 ± 0.7 |
| 1 | 1475.0 | -7.0 ± 0.7 | -11.3 ± 0.7 |
| 1 | 1472.5 | -5.8 ± 0.7 | -13.7 ± 0.7 |
| 1 | 1470.0 | -4.9 ± 0.7 | -15.8 ± 0.7 |
| 2 | 1500.0 | 7.2 ± 0.7 | 11.1 ± 0.7 |
| 2 | 1497.5 | 9.5 ± 0.7 | 9.5 ± 0.7 |
| 2 | 1495.0 | 10.0 ± 0.7 | 7.8 ± 0.7 |
| 2 | 1492.5 | 11.2 ± 0.7 | 5.8 ± 0.7 |
| 2 | 1490.0 | 12.4 ± 0.7 | 3.0 ± 0.7 |
| 2 | 1487.5 | 12.7 ± 0.7 | 0.5 ± 0.7 |
| 2 | 1485.0 | 12.0 ± 0.7 | -1.3 ± 0.7 |
| 2 | 1482.5 | 11.1 ± 0.7 | -3.3 ± 0.7 |
| 2 | 1480.0 | 9.9 ± 0.7 | -6.3 ± 0.7 |
| 2 | 1477.5 | 8.7 ± 0.7 | -8.6 ± 0.7 |
| 2 | 1475.0 | 7.0 ± 0.7 | -10.7 ± 0.7 |
| 2 | 1472.5 | 5.0 ± 0.7 | -12.3 ± 0.7 |
| 2 | 1470.0 | 4.0 ± 0.7 | -15.9 ± 0.7 |
