# Supplementary material for: Automated verification of source dwell position in lunar ovoid applicators
Source: J Appl Clin Med Phys. 2026 Oct 30;27(11):e70887. doi: 10.1002/acm2.70887 (PMC13722779; doi:10.1002/acm2.70887)
Supplement: Supplementary file 5 [file ACM2-27-e70887-s005.docx]

**Table S-5.** Distances between the manufacturer’s libraries and the most distant autoradiograph centroid, $d_{max}$, in the case of multiple exposures depicted in Fig. 4 (c, d) from the main manuscript. The afterloader channel and position are also included.

|  |  | **22 mm ovoids** | | **26 mm ovoids** | |
| --- | --- | --- | --- | --- | --- |
| **Position (mm)** | **Afterloader channel** | $d_{max}$ **(mm)** | Num. exposures | $d_{max}$ **(mm)** | Num. exposures |
| 1500.0 | 1 | 2.5 ± 1.1 | 8 | 2.9 ± 1.1 | 13 |
| 1490.0 | 1 | 1.6 ± 1.0 | 8 | 2.0 ± 1.1 | 8 |
| 1480.0 | 1 | 2.0 ± 1.0 | 6 | 2.2 ± 1.1 | 8 |
| 1470.0 | 1 |  |  | 2.9 ± 0.9 | 4 |
| 1500.0 | 2 | 1.3 ± 1.1 | 8 | 2.0 ± 1.0 | 14 |
| 1497.5 | 2 | 1.4 ± 1.1 | 7 | 1.9 ± 1.1 | 8 |
| 1487.5 | 2 | 1.2 ± 1.1 | 8 | 1.8 ± 0.9 | 8 |
| 1477.5 | 2 | 1.7 ± 0.8 | 3 | 1.3 ± 1.1 | 5 |
