# Supplementary material for: Automated verification of source dwell position in lunar ovoid applicators
Source: J Appl Clin Med Phys. 2026 Oct 30;27(11):e70887. doi: 10.1002/acm2.70887 (PMC13722779; doi:10.1002/acm2.70887)
Supplement: Supplementary file 6 [file ACM2-27-e70887-s006.docx]

**Table S-1.** Fabrication parameters for the design of the Venezia^TM^ holder.

| **Settings for the 3D printer** | |
| --- | --- |
| Infill density | 20 % |
| Shell thickness | 0.8 mm |
| Layer height | 1.0 mm |
| Material | Generic PLA |
| Total print time | 2 hours 44 minutes |
| Filament amount | 18 g |
| Support material | Yes |
| Support type | Tree |
| Support placement | If possible, in non-functional surfaces |
